# Firdausi Qadri: the struggle against cholera

**DOI:** 10.2471/BLT.23.030423

**Published:** 2023-04-01

## Abstract

Firdausi Qadri talks to Gary Humphreys about the need for new cholera vaccines and increased vaccine production to meet growing regional and global demand.

*Q: You are best known for your work on cholera. Has that always been your principal focus?*

A: Living in the Ganges Delta and growing up in a country which sees at least 300 000 severe cholera cases each year, what the pathogen does to the human body has certainly been a preoccupation of mine for a long time. However, I have worked on many other pathogens and diseases, including enterotoxigenic *Escherichia coli* and *Helicobacter pylori,* and when I first joined the icddr,b, I was actually working on *Shigella*. This was in the late 1980s, and the ‘higher-ups’ felt that we had viable treatments for cholera and so I should focus my attention elsewhere. But then, two years into my time here, the hospital on the ground floor started filling up with patients presenting with a cholera-like disease which had everyone puzzled. On closer inspection, testing with available reagents, we realized that it was not El Tor biotype O1; it was a different serogroup that was later labelled variant O139. In the following years, O139 became one of the two serogroups that caused serious outbreaks. Since then, it has faded away, only showing up in a few sporadic cases, but it was the bug that changed my career.

*Q: In what sense?*

A: Well, I had reached something of a dead end with *Shigella*. There were so many people working on it that it was difficult to make a meaningful contribution. When O139 arrived, that all changed; there were so many questions to be answered, questions about the antibody response, the mucosal immune responses, the protective immune responses, etc. And at the same time, new technologies ranging from flow cytometry to antibody secreting cell analysis were becoming available that made it possible to see how the organism behaved inside the human body. Genomics was also taking off. So, I really threw myself into the research, using all these new tools and writing papers.

And then one day I was sitting in my lab, and I became aware of the cholera patients crying out downstairs. At that time, I was down on the second floor and the hospital was just below me. We have two seasonal outbreaks in Bangladesh, so it wasn’t something I had never heard before, but for some reason it came to me that it was all very well doing this clever laboratory work and contributing to scientific journals and so on, but I was not directly helping anyone. That was when I started my work on oral cholera vaccines – how to make the best use of them, how to make them accessible to people and how to support their development. It has been a long, long road but here I am, now up on the eighth floor, and still working on cholera vaccine optimization and access, with much of my recent work focusing on affordable cholera vaccines including Cholvax, an inactivated, whole-cell oral vaccine which is manufactured here in Bangladesh.

“[WHO-approved oral cholera vaccines] are not produced in sufficient quantities to meet global demand.”

*Q: There are three oral cholera vaccines approved for use by the World Health Organization (WHO). Why do we need another?*

A: Well, first because only two of those vaccines, Shanchol and Euvichol, are available for mass vaccination campaigns. On top of that, they cost around 3 and 4 United States dollars (US$) for two doses for full immunization, and are not produced in sufficient quantities to meet global demand. The situation got worse at the end of 2022 when the Indian subsidiary of Sanofi stopped production of Shanchol. The problem we have is that cholera vaccine production is not a lucrative activity and so private sector companies are reluctant to get behind it. It was because of supply concerns that the ICG (International Coordinating Group - the body that manages emergency supplies of vaccines) temporarily suspended the standard two-dose vaccination regimen used in cholera outbreak response in October of last year, favouring a single-dose approach.

*Q: Is the switch to a one-dose regime a matter of concern in your opinion?*

A: I understand the reasoning behind it, but it is less than ideal. According to our research, a single dose confers significant protection for adults and children above 5 years of age, but children below that age remain largely exposed. To address this challenge, we need to be able to ramp up vaccine production and develop new oral vaccines that are affordable, effective and suitable for mass distribution in what are often challenging circumstances. We estimate that we will need around 180 million doses of oral cholera vaccine just to control the disease in Bangladesh in the next five years. We have been supporting efforts to meet that need, notably with the development of Cholvax.

*Q: Can you tell me a little more about the vaccine?*

A: It was developed in a public-private partnership that we initiated between the International Vaccine Institute and vaccine manufacturers here, and is now being produced by Incepta Vaccine Ltd, thanks to a technology transfer from the International Vaccine Institute in Seoul, Republic of Korea. Between 2016 and 2017, the icddr,b conducted a randomized clinical trial comparing it to Shanchol in just over 2000 healthy adults and children here in the city. The results indicated comparable immunogenicity and safety to Shanchol. Cholvax is registered in Bangladesh and is currently selling at 1000 Bangladeshi Taka (US$9.50) per dose.

*Q: Is it available outside Bangladesh?*

A: It is currently available commercially, but has yet to be WHO prequalified, so cannot be purchased or recommended for mass immunization campaigns by UN (United Nations) agencies or the likes of Gavi, (Gavi, the Vaccine Alliance), or the GTFCC (Global Task Force for Cholera Control). So its impact on the global vaccine landscape has so far been limited.

*Q: What is holding up WHO prequalification?*

A: The absence of a NRA (National Regulatory Authority) for vaccines and drugs, which is one of the criteria for WHO prequalification. The legislation required to establish a NRA is currently moving through parliament and once it has been approved, a WHO team will be coming to Bangladesh to conduct the final assessments. There have been many delays, but I am optimistic that we will have some good news from parliament in the coming months. I told my staff that the day the legislation is passed, I will buy everyone sweets.

*Q: Why has it taken parliament so long to pass legislation?*

A: The regulation itself is fairly complex, covering a range of activities and inputs, and the COVID-19 (coronavirus disease 2019) pandemic hasn’t helped. That said, it is in part because of COVID-19 that we are seeing some movement now. We were all reminded of how important it is for lower-income countries to have their own vaccine development and production capacity during the pandemic, especially when the third COVID-19 wave hit us between June and August 2021. So the mood has changed, if you like, and we are already seeing concrete improvements.

“I became aware of the cholera patients crying out downstairs.”

*Q: Can you give an example?*

A: There has been a big expansion in biomedical laboratory capacity in the country, including labs equipped for genomic sequencing. We went from 10 biomedical labs in 2020 to 500 labs now, around 15 of which can conduct genomic sequencing. They made a significant contribution to SARS-CoV-2 (severe acute respiratory syndrome coronavirus 2) screening during the pandemic. Much more needs to be done of course, notably in terms of vaccine production capacity. We have the facilities in Bangladesh, two of which can meet and indeed surpass national demand, but a separate bigger facility will be needed if we are to supply the likes of Gavi and UNICEF (United Nations Children’s Fund). Based on current trends the demand for reactive vaccination alone is only going to increase and so we need to be ready.

*Q: The icddr,b also developed a rapid diagnostic test (RDT) for cholera that is suitable for use in resource-poor countries. Has that test been evaluated in the field?*

A: Indeed, it has. We in fact developed it in collaboration with a local company and field tested it in 2017. The trial indicated a sensitivity of 76% which is superior to products on the market. The test is robust and simple, comprising a dipstick that is dipped into a tube with a stool specimen, and provides a qualitative result in the form of a coloured band that indicates the presence of the O1 serogroup within a maximum of 15 minutes. It is the first and only cholera RDT to be developed in Bangladesh, and I hope to see it go into mass production soon.

Raising the visibility of cholera is not just a matter of improving access to diagnostics, however; it also depends on tackling stigma. There is a burden of shame associated with the disease which hampers effective communication and leads to underreporting and inappropriate treatment. Here at the icddr,b we have cholera downstairs, we have cholera in the city, we have cholera everywhere, but there is still a reluctance to talk about it. Things are improving and fortunately the government is showing the way. I hope that going forward attitudes will continue to change towards this ancient disease, which still imposes such a burden of suffering on the modern world.

*Q: You mention being up on the eighth floor of the icddr,b building now, where presumably you are focused on your directorial duties. Do you ever miss your microscope?*

A: Well fortunately, I am just a few steps away from my laboratory up here. So, I've been able to keep very much in touch with that even though it is true that my directorial duties keep me very busy. I’m also very connected with the hospital downstairs and do a lot of work on capacity building, supporting the development of young scientists and researchers both in the lab and in the field. I am also just a short distance from places like Cox's Bazar and Chittagong, where we are carrying out our field research on surveillance and vaccine campaigns. That said, I think at heart I am a pathogen person, and still find immense pleasure when I do look down a microscope or analyse new data emerging from the work of my colleagues. It is just that these days, I cannot do either without being acutely conscious of the world outside and worrying about how my work will benefit Bangladesh.

